# Neuroprognostication after cardiac arrest in patients without withdrawal of life-sustaining therapy: a prospective observational multicenter study

**DOI:** 10.1186/s13054-026-06209-0

**Published:** 2026-07-24

**Authors:** Danae Goetze, Jan Rémi, Christoph Leithner, Victoria Diethei, Vincent Fleischhauer, Bastian Pauli, Bernadette Einhaeupl, Tina Luther, Martin Rosenfelder, Alex Lopez-Rolon, Stefan Czermak, Matthias W. A. Angstwurm, Suzette Heck, Bernhard Zwissler, Hanns Lohner, Ana-Lioara Arva, Thomas Pfefferkorn, Andreas Blüthgen, Markus Naumann, Philip Raake, Hauke Schneider, Rüdiger Ilg, Friedemann Müller, Jürgen Herzog, Katrin Rauen, Ralf Strobl, Daniela Koller, Eva Grill, Andreas Straube, Andreas Bender

**Affiliations:** 1 Neurorehabilitation, Faculty of Medicine, University of Augsburg, Augsburg, Germany; 2 Institute for Medical Information Processing, Biometry, and Epidemiology (IBE), LMU Medizin, Ludwig-Maximilians-Universität München, Pettenkofer School of Public Health, Munich, Germany; 3 Department of Neurology, LMU University Hospital, LMU Munich, Munich, Germany; 4 Department of Neurology and Experimental Neurology, Charité – Universitätsmedizin Berlin, Corporate Member of Freie Universität Berlin and Humboldt-Universität zu Berlin, Berlin, Germany; 5 Therapiezentrum Burgau, Hospital for Neurological Rehabilitation, Burgau, Germany; 6 Klinik für Neurologie, München Klinik Harlaching, Munich, Germany; 7 Department of Medicine IV, LMU University Hospital, LMU Munich, Munich, Germany; 8 Klinik für Anästhesiologie, Klinikum der Universität München, LMU München, Munich, Deutschland; 9 Neurologische Klinik, RoMed Klinikum Rosenheim, Rosenheim, Germany; 10 Department of Neurology, Klinikum Ingolstadt, Ingolstadt, Germany; 11 Department of Internal Medicine I, University Hospital Augsburg, University of Augsburg, Augsburg, Germany; 12 Department of Neurology and Clinical Neurophysiology, University Hospital Augsburg, Augsburg, Germany; 13 Department of Neurology, University Hospital Augsburg, Augsburg, Germany; 14 Department of Neurology, Klinikum rechts der Isar, Technische Universität München, Munich, Germany; 15 Asklepios Stadtklinik, Bad Tölz, Germany; 16 Schön Klinik Bad Aibling, Bad Aibling, Germany; 17 Schön Klinik Munich- Schwabing, Munich, Germany; 18 Psychiatric University Hospital Zurich & Neuroscience Center Zurich (ZNZ), Zurich, Switzerland; 19 Institute for Medical Information Processing, Biometry, and Epidemiology (IBE), LMU Medizin, Ludwig-Maximilians-Universität München, Munich, Germany; 20 Department of Orthopaedics and Trauma Surgery, Musculoskeletal University Center Munich (MUM), University Hospital, LMU Munich, Munich, Germany

**Keywords:** Cardiac arrest, Neuroprognostication, Withdrawal of life-sustaining therapy, Disorders of consciousness, Multimodal prognostication, Outcome prediction

## Abstract

**Background:**

Neuroprognostication after cardiac arrest (CA) is critical for guiding treatment decisions. However, concerns about self-fulfilling prophecy related to withdrawal of life-sustaining therapy (WLST) complicate outcome prediction. This study evaluated the accuracy of guideline-recommended prognostic markers within 14 days after CA for predicting poor 12-month outcomes in patients without WLST during the first four weeks after CA.

**Methods:**

This prospective multicenter observational study enrolled adults who remained comatose 72 h after CA across eight German hospitals between 2014 and 2017. Patients with WLST during the first four weeks, stroke, pre-existing disorders of consciousness, or terminal malignancy were excluded, leaving 101 patients for analysis. Prognostic markers assessed included pupillary light and corneal reflexes (PLR + CR), EEG, somatosensory evoked potentials (SEP), neuron-specific enolase (NSE) concentration, and the best Coma Recovery Scale–Revised (CRS-R) score. Poor outcome was defined as a modified Rankin Scale score of 4–6 at 12 months.

**Results:**

Poor outcomes occurred in 67.3% of patients. Individual markers demonstrated high specificity but limited sensitivity, with variable false-positive rates (FPRs). Repeated assessments and the combination of two or more unfavorable markers markedly reduced FPRs. Within 14 days, the presence of two or more unfavorable markers yielded no false positives and was strongly associated with poor outcome (adjusted OR 34.7). Adding the best CRS-R score to established markers significantly improved prognostic discrimination (AUC 0.925 vs. 0.888).

**Conclusions:**

In patients who remained comatose three days after CA and in whom multimodal prognosis becomes clinically relevant, guideline-recommended assessment identified poor-outcome patients with high specificity and consistency with prior multimodal prognostication cohorts. Although self-fulfilling-prophecy bias cannot be fully excluded in any observational prognostication cohort, the marker profiles of WLST-excluded patients within the first four weeks matched or exceeded those of patients with documented poor outcomes and did not resemble those of recoverable patients, which reduces - though it does not completely rule it out - the likelihood that such bias is the primary reason for the observed associations. The best CRS-R score provided additional prognostic value, particularly in cases of uncertain prognosis, supporting its integration into multimodal assessment frameworks.

**Trial registration:**

ClinicalTrials.gov: NCT02231060. Registered: August 2014. First patient enrolled: October 2014.

**Supplementary Information:**

The online version contains supplementary material available at 10.1186/s13054-026-06209-0.

## Background

Cardiac arrest (CA) represents a major public health burden with one-year survival rates below 10% after hospital discharge [1, 2]. In successfully resuscitated patients, hypoxic-ischemic brain injury (HIBI) represents an important determinant of neurological outcome, potentially causing disorders of consciousness (DoC) [3, 4]. Although HIBI is responsible for most deaths following CA, only a minority meet the criteria for brain death [3]. Most deaths occur after withdrawal of life-sustaining therapy (WLST) based on a poor prognosis, raising concerns that prognostication not only predicts death but contributes to a self-fulfilling prophecy [5]. As most patients with good outcome regain consciousness within 3–4 days, prognostic uncertainty mainly concerns those who remain comatose beyond this time point [5]. Therefore delaying prognostication until at least 72 h after CA is recommended [5–8].

To support reliable prognostication, international guidelines recommend a multimodal approach integrating clinical examination, electrophysiology, biomarkers, and neuroimaging [6–8]. Accordingly, this study assessed brainstem function by pupillary light and corneal reflex (PLR + CR), cortical function by EEG, thalamocortical connectivity by somatosensory evoked potentials (SEP), and neuronal injury by neuron-specific enolase (NSE) concentration [9].

Given the established prognostic value of structured behavioral assessment in prolonged DoC [10, 11], we additionally evaluated whether the best Coma Recovery Scale–Revised (CRS-R) score within 14 days provided incremental prognostic value beyond these markers.

We hypothesized that multimodal prognostic markers would exhibit high specificity (false-positive rate (FPR) < 10%) for poor 12-month outcomes in a cohort of patients who were in a coma for at least 72 h following CA and were followed up for the first four weeks without WLST, and that in patients with unclear marker profiles, the absence or presence of behavioral recovery within the first two weeks, as assessed by the CRS-R, might be precisely the signal that distinguishes patients with the capacity for functional improvement from those without.

## Methods

This multicenter prospective observational study was conducted at eight German hospitals between January 1, 2014, and December 31, 2017. Data were collected according to Utstein-style recommendations [12] by trained study personnel. The study was approved by the ethics committee of University of Munich, LMU, Germany (no. 362 − 14). Written informed consent was obtained from legal representatives. The trial was registered at ClinicalTrials.gov (NCT02231060). The trial was originally designed with a Simon two-stage stopping rule (planned total *n* = 172, interim analysis at *n* = 72), as detailed in the published study protocol [13]. This design was intended primarily for feasibility and safety assessment; the diagnostic-accuracy and multivariable analyses reported here are exploratory in nature with respect to the performance of guideline-recommended prognostic markers.

### Participants

Individuals who remained comatose three days after CA despite cessation of sedatives (as sedation is a significant confounding factor in neuroprognostic assessment, according to guidelines [6–8]) were included using the following criteria: (i) age 18–85 years; (ii) CA as cause of intensive care unit (ICU) admission; (iii) Glasgow Coma Scale (GCS) < 8 at enrollment; (iv) informed consent from a legal guardian. Exclusion criteria were: (i) stroke; (ii) pre-existing DoC; (iii) terminal malignancy; (iv) advance directives requesting WLST or WLST during ICU care. All patients received guideline-based post CA care. Since the prognostic tests were not conducted solely as part of a study but are part of routine clinical practice, the results could not be withheld from either the treating clinical teams or the patients’ families; the decision to change treatment goals therefore remained with the treating team and the families. WLST decisions were not influenced by participation in the study and were made on clinical and ethical grounds. In our context, WLST decisions for patients with similar prognoses are primarily determined by factors other than prognostic markers alone (most notably by documented or presumed patient wishes expressed through advance directives and discussions with representatives or family members, by premorbid functional status, and by the burden of non-neurological comorbidities). Patients undergoing WLST within the first four weeks (28 days) after CA (hereafter referred to as early WLST) were excluded. This window was chosen to bracket the period in which prognostication-related WLST is most frequent yet, according to current guidelines, least justified: most WLST decisions are made within approximately 3–10 days,^14,15^ whereas delayed recovery of consciousness up to two weeks or longer is well documented [16, 17] and guidelines on DoC advise against presuming a universally poor prognosis within the first 28 days after injury [18].

### Prognostic investigations

Prognostic markers were recorded upon study enrollment and dichotomized as favorable or unfavorable according to guideline-based thresholds for poor prognosis [6–8]: (i) PLR + CR: bilateral absence; (ii) EEG: presence of highly malignant patterns (suppressed background ± discharges; burst-suppression ± discharges)^19^; (iii) SEP: bilateral absence of the cortical N20 wave after median nerve stimulation; (iv) NSE: serum concentrations ≥ 90 µg/l [8].

PLR + CR was assessed using the brainstem reflex subscale of the Full Outline of UnResponsiveness (FOUR) score, with scores ≤ 1 defined as absent PLR + CR; the assessment was performed by trained study personnel. EEGs were reviewed by two neurologists blinded to clinical and prognostic data and classified by consensus according to standardized terminology (highly malignant, malignant, benign) [19]. SEP status was based on routine reports. Recordings reported as bilaterally absent were reassessed by four blinded neurologists. Non-evaluable recordings were excluded according to predefined methodological criteria: (i) performance of peripheral, spinal, and cortical recordings per side; (ii) bilateral reproducibility of spinal potentials (N13a); (iii) noise level below 0.25 µV in all cortical recordings [20].

The CRS-R (range 0–23) is the gold standard for behavioral assessment in DoC, allowing standardized diagnosis across all DoC levels [21]. Initial and longitudinal CRS-R scores within the first month after brain injury can predict functional outcome [10, 11].

### Outcome measures

The primary endpoint was neurological outcome at 12 months, assessed with the modified Rankin Scale (mRS) and dichotomized as good (0–3) or poor (4–6). The mRS was conducted by the study personnel using a hierarchical assessment strategy. Where feasible, patients were visited at home, allowing in-person administration of the mRS together with the CRS-R and other functional assessments. If a home visit was not possible, outcome was assessed by structured telephone interview; where neither in-person nor telephone contact could be arranged, a structured questionnaire was completed by mail. At most participating centers, outcome assessors were independent of the in-hospital study team and blinded to predictor results; at one center, the same investigators contributed to enrollment-phase data collection and to 12-month outcome assessment, and were therefore not blinded for that subset of patients. Baseline data included demographics, comorbidities, CA-related variables, and neurological assessments (GCS, FOUR, CRS-R). CRS-R, GCS, and FOUR were assessed repeatedly during the first two weeks and at 12 months after CA. Prognostic markers were obtained within 7 and 14 days. Functional independence was assessed using the Barthel Index. Assessment time points are summarized in Supplementary Table S1.

### Statistical analysis

Categorical variables are reported as counts (%) and continuous variables as mean with standard deviation (SD) or median with interquartile range (IQR). Baseline characteristics were compared between outcome groups using Fisher’s exact or Wilcoxon rank-sum test.

Marker performance was evaluated within 7- and 14-days using sensitivity, specificity, predictive values, and FPR based on the last available measurement. Additional analyses compared first and last available marker assessments. In a sensitivity analysis, we compared WLST patients (*n* = 39) with the two outcome subgroups of the analyzed cohort (good and poor) for all baseline, marker, and CRS-R variables using pairwise Wilcoxon or Fisher exact tests with Bonferroni correction across three pairwise contrasts. Further, we applied the multivariable Firth model, which was refit on the analyzed cohort and therefore independent of WLST patients’ actual outcomes to WLST patients with complete predictor data (*n* = 30), reporting the distribution of predicted poor-outcome probabilities and the proportions above clinically relevant thresholds (≥ 0.5, ≥ 0.8, ≥ 0.9).

Markers were categorized as 0, 1, or ≥ 2 unfavorable markers categories and related to poor outcome using age-adjusted Firth logistic regression. Patients with at least two different measured markers in the respective time window were included. Shifts in marker category between ≤ 7 and ≤ 14 days were examined using a transition analysis, including all patients with at least two different measured markers at ≤ 7 days.

The discriminative ability of best CRS-R within 14 days was assessed by receiver operating characteristic (ROC) analysis with area under the curve (AUC) and 95% confidence intervals (CI). In a systematic analysis, the CRS-R cutoffs were defined as those that exhibited the highest sensitivity among values with a specificity of ≥ 0.90. To quantify the incremental value of CRS-R beyond week-1 marker results, a two-step classification approach was applied: marker category at ≤ 7 days first, and best CRS-*R* ≤ 14 days second; net gain (correctly minus incorrectly additionally classified patients) was reported. Best CRS-R within 14 days was analyzed in multivariable Firth models extending a baseline model with age, EEG, PLR + CR, and NSE (scaled per 10 µg/L). In a systematic review age was described as the most frequently included pre-arrest factors affecting clinical outcome after attempted resuscitation and was therefore added to the model [22]. Due to high missingness SEP was excluded from models. SEP missingness within 7 and 14 days post-CA is not outcome-associated (*p* = 0.73 / 1.0) or with any other predictor, age or comorbidity (mCIRS score) (all *p* ≥ 0.05), supporting the assumption of missingness at random for complete case analyses. Incremental value was tested by penalized likelihood-ratio test. Model performance was assessed by AUC, Brier score, and calibration, with bootstrap optimism correction and repeated cross-validation. To address the possibility that the prognostic value of “best CRS-R within 14 days” was confounded by survival time we performed a 2 × 2 sensitivity design varying the CRS-R measurement window (best score within 7 days vs. within 14 days) and the survival restriction (full analyzed cohort vs. patients surviving at least 14 days post-CA). The extended multivariable Firth logistic regression model was refit in each of the four cells using identical predictor transformations as in the primary analysis. Three pre-specified pairwise comparisons separated the contributions of survival opportunity from those of recovery dynamics: (i) within the 7-day measurement window, full cohort vs. ≥14-day survivors; (ii) within the 14-day measurement window, full cohort vs. ≥14-day survivors; and (iii) within the ≥ 14-day-survivor subsample, 7-day vs. 14-day measurement window. To assess the potential impact of complete-case analysis on the 9 patients lost to 12-month follow-up, (i) a comparison of their baseline characteristics, marker, and CRS-R characteristics to the analysed cohort and (ii) a repeated primary marker-performance and Firth burden analyses under the assumption that all lost to follow-up patients had an entirely favorable (mRS 0) or entirely unfavorable (mRS 6) 12-month outcome was performed.

Odds ratios (ORs) with 95% CIs and two-sided p-values were reported. All analyses were performed in R (version 4.5.1) using ggplot2, logistf, pROC, ggalluvial and rsample.

## Results

### Study population

**Fig. 1:**
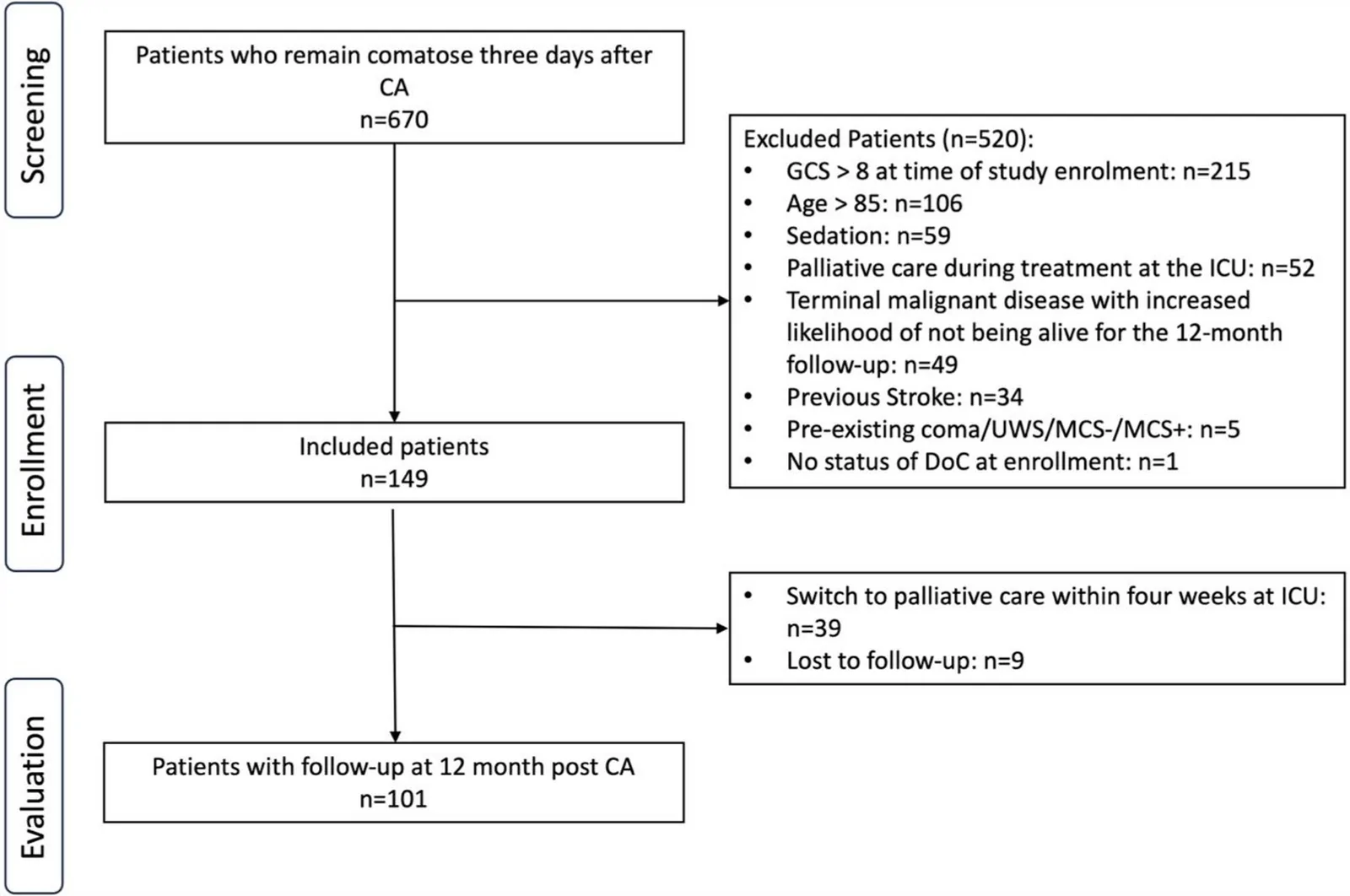
Flowchart of eligible patients included in the analysis

Of 670 patients screened, 149 patients met the primary inclusion criteria; Fig. 1. The final analysis included 101 patients. Median age was 63 (54–74) years, and 72 patients (71.3%) were male

At 12 months, 33 patients (32.7%) had good outcomes, including mRS 0 in 8 (24.2%), mRS 1 in 11 (33.3%), mRS 2 in 9 (27.3%), and mRS 3 in 5 patients (15.2%). Poor outcome was observed in 68 patients (67.3%), including mRS 4 in 9 (13.2%), mRS 5 in 16 (23.5%), and mRS 6 (representing deceased patients) in 43 patients (63.2%; mean survival was 95.2 [104.5]). Eight patients (19.0%) died after more than six months. Nine patients (8.2% of the cohort) were excluded because of missing 12-month mRS (lost to follow-up); their baseline, marker, and CRS-R characteristics did not differ significantly from the analysis cohort (all *p* ≥ 0.19).

Baseline demographic and arrest-related characteristics were comparable between outcome groups; Table 1. Follow-up CRS-R, GCS, FOUR, and BI scores were lower in the poor outcome group (all *p* < 0.001). Within 14 days, unfavorable PLR + CR and highly malignant EEG patterns were more frequent, and NSE concentrations were higher, in poor outcome patients.

**Table 1:** Demographic and clinical characteristics of patients at time of enrollment (baseline [BL]) and 12-months follow-up (FU)

Variable	Overall (*N* = 101)	Good outcome (*N* = 33)	Poor outcome (*N* = 68)	*P* value	
Age (years), median (IQR)	63 (54–74)	60 (50–71)	65.5 (56–75)	0.1	
Sex, n (%)				0.163	
Female	29 (28.7)	6 (18.2)	23 (33.8)		
Male	72 (71.3)	27 (81.8)	45 (66.2)		
Location of cardiac arrest, n (%)				1	
OHCA	65 (72.2)	22 (73.3)	43 (71.7)		
IHCA	25 (27.8)	8 (26.7)	17 (28.3)		
Missings	n_miss_=11 (10.9)	n_miss_ =3 (9.1)	n_miss_ =8 (11.8)		
Cause of cardiac arrest, n (%)				0.167	
Cardiac	63 (70.8)	25 (83.3)	38 (64.4)		
Traumatic	2 (2.2)	1 (3.3)	1 (1.7)		
Hypoxic	15 (16.9)	1 (3.3)	14 (23.7)		
Other	7 (7.9)	2 (6.7)	5 (8.5)		
Missings	n_miss_ =14 (13.9)	n_miss_ =4 (12.1)	n_miss_ =10 (14.7)		
BL mCIRS score, median (IQR)	6 (3.5–10.5)	4.5 (2-6.25)	8 (4-11.5)	0.004	
Missings	n_miss_ =2 (2.0)	n_miss_ =1 (3.0)	n_miss_ =1 (1.5)		
LOS ICU (days), median (IQR)	16 (11–30)	16 (11–33)	15 (11–28)	0.928	
Missings	n_miss_ =39 (38.6)	n_miss_ =13 (39.4)	n_miss_ =26 (38.2)		
tCPR (minutes), median (IQR)	20 (10–30)	12 (9–23)	21 (11–36)	0.092	
Missings	n_miss_ =52 (51.5)	n_miss_ =17 (51.5)	n_miss_ =35 (51.5)		
tROSC (minutes), median (IQR)	30 (22–45)	30 (15–45)	30 (23–41)	0.772	
Missings	n_miss_ =71 (70.3)	n_miss_ =19 (57.6)	n_miss_ =42 (61.8)		
LOS Neurorehabilitation (days), median (IQR)	59 (29–99)	35 (20–71)	61 (31–109)	0.174	
Missings	n_miss_ =57 (56.4)	n_miss_ =23 (69.7)	n_miss_ =34 (50.0)		
Death during ICU stay, n (%)	1 (1.6)	0 (0.0)	1 (2.4)	1	
Initial CRS-R score ≤ 14 days, median (IQR)	1 (0-3.25)	1 (0–3)	1 (0-3.5)	0.423	
Missings	n_miss_ =1 (1.0)	n_miss_ =0 (0.0)	n_miss_ =1 (1.5)		
Best CRS-R score ≤ 14 days, median (IQR)	4.5 (2–17)	17 (5–23)	4 (1–6)	< 0.001	
Missings	n_miss_ =1 (1.0)	n_miss_ =0 (0.0)	n_miss_ =1 (1.5)		
Median CRS-R score ≤ 14 days, median (IQR)	3.5 (1.5-10.25)	9.5 (3.5–13)	2 (1-5.75)	< 0.001	
Missings	n_miss_ =1 (1.0)	n_miss_ =0 (0.0)	n_miss_ =1 (1.5)		
FU CRS-R score, median (IQR)	23 (22–23)	23 (23–23)	13 (7–23)	< 0.001	
Missings	n_miss_ =47 (46.5)	n_miss_ =0 (0.0)	n_miss_ =47 (69.1)		
BL GCS, median (IQR)	3 (3–5)	3 (3–5)	3 (3–6)	0.246	
Missings	n_miss_ =0 (0.0)	n_miss_ =0 (0.0)	n_miss_ =0 (0.0)		
FU GCS score, median (IQR)	15 (15–15)	15 (15–15)	14 (8–15)	< 0.001	
Missings	n_miss_ =47 (46.5)	n_miss_ =0 (0.0)	n_miss_ =47 (69.1)		
BL FOUR score, median (IQR)	5 (3–7)	4 (4–6)	5 (3–7)	0.403	
Missings	n_miss_ =1 (1.0)	n_miss_ =0 (0.0)	n_miss_ =1 (1.5)		
FU FOUR score, median (IQR)	16 (16–16)	16 (16–16)	14 (12–16)	< 0.001	
Missings	n_miss_ =47 (46.5)	n_miss_ =0 (0.0)	n_miss_ =47 (69.1)		
FU Barthel Index, median (IQR)	95 (12.5–100)	100 (100–100)	10 (5–25)	< 0.001	
Missings	n_miss_ =47 (46.5)	n_miss_ =0 (0.0)	n_miss_ =47 (69.1)		
Unfavorable PLR + CR ≤ 7 days, n/N (%)	24/83 (28.9)	5/29 (17.2)	19/54 (35.2)	0.086	
Time of measurement, median (IQR)	5 (3–6)	4 (3–6)	5 (4–7)		
EEG pattern ≤ 7 days, n/N (%)				0.042	
Benign	18/59 (30.5)	9/19 (47.4)	9/40 (22.5)		
Malignant	23/59 (39.0)	8/19 (42.1)	15/40 (37.5)		
Highly malignant	18/59 (30.5)	2/19 (10.5)	16/40 (40.0)		
Time of measurement, median (IQR)	5 (3–7)	4 (2–4)	6 (4–8)		
Unfavorable SEP ≤ 7 days, n/N (%)	2/45 (4.4)	0/16 (0.0)	2/29 (6.9)	0.531	
Time of measurement, median (IQR)	5 (4–7)	4 (3–5)	5 (4–8)		
NSE ≤ 7 days, mean (SD)	50.59 (55.36)	28.29 (15.20)	61.57 (64.13)	< 0.001	
Time of measurement, median (IQR)	3 (2–4)	3 (2–4)	3 (2–5)		
Missings	n_miss_ =4 (4.0)	n_miss_ =1 (3.0)	n_miss_ =3 (4.4)		
Unfavorable PLR + CR ≤ 14 days, n/N (%)	17/98 (17.3)	1/33 (3.0)	16/65 (24.6)	0.008	
Time of measurement, median (IQR)	10 (9–12)	10 (9–11)	11 (9–12)		
EEG pattern ≤ 14 days, n/N (%)				< 0.001	
Benign	28/75 (37.3)	13/20 (65.0)	15/45 (27.3)		
Malignant	23/75 (30.7)	7/20 (35.0)	16/45 (29.1)		
Highly malignant	24/75 (32.0)	0/20 (0.0)	24/45 (43.6)		
Time of measurement, median (IQR)	11 (10–13)	10 (9–11)	11 (10–13)		
Unfavorable SEP ≤ 14 days, n/N (%)	3/61 (4.9)	0/20 (0.0)	3/41 (7.3)	0.544	
Time of measurement, median (IQR)	11 (10–13)	10 (10–11)	12 (11–14)		
NSE ≤ 14 days, mean (SD)	38.40 (36.12)	25.94 (14.97)	44.53 (41.62)	0.002	
Time of measurement, median (IQR)	9 (6–11)	10 (7–10)	9 (6–11)		
Missings	n_miss_ =1 (1.0)	n_miss_ =0 (0.0)	n_miss_ =1 (1.5)		

BI, Barthel Index; BL, Baseline (study enrollment); CA, Cardiac arrest; CRS-R, Coma Recovery Scale-Revised; FOUR, Full Outline of UnResponsiveness; FU, Follow-Up; GCS, Glasgow Coma Scale; ICU, Intensive Care Unit; IHCA, In-Hospital Cardiac Arrest; LOS, Length of Stay; mCIRS, Modified Cumulative Illness Rating Scale; mRS, Modified Rankin Scale; n_miss_, number of missing values (for CRS-R, GCS, FOUR and BI at FU missing values include patients (*n* = 43) who died before the respective assessment and therefore had no measurable value); NSE, neuron-specific enolase; OHCA, Out-of-hospital cardiac arrest; PLR + CR, pupillary light and corneal reflex; SEP, somatosensory evoked potentials N20 wave; tCPR, time to begin of Cardiopulmonary Resuscitation; tROSC, time to Return of Spontaneous Circulation

### Prognostic performance of single and paired markers

Single unfavorable markers showed low to moderate sensitivity but generally high specificity for poor outcome; Table 2. EEG had the highest sensitivity within 7 (0.40, 95% CI 0.25–0.57) and 14 days (0.44, 95% CI 0.30–0.58), whereas SEP and NSE were specific but poorly sensitive. Specificity increased over time, with no FP classifications within 14 days for EEG, SEP, and NSE and one for PLR + CR (FPR 3%). Combinations of two unfavorable markers were highly reliable when present (specificity > 93%) with no FPRs within 14 days but captured only a minority of patients with poor outcome (sensitivity < 22%).

**Table 2:** Prognostic performance of single and paired prognostic markers within 7 and 14 days after CA

Prognostic marker (single and paired)	*n*	TP	FP	FN	TN	Sensitivity (95% CI)	Specificity (95% CI)	PPV (95% CI)	NPV (95% CI)	FPR (%)
**Assessments ≤ 7 after CA**										
PLR + CR	83	19	5	35	24	0.35 (0.23–0.49)	0.83 (0.64–0.94)	0.79 (0.58–0.93)	0.41 (0.28–0.54)	17.2
EEG	59	16	2	24	17	0.40 (0.25–0.57)	0.89 (0.67–0.99)	0.89 (0.65–0.99)	0.41 (0.26–0.58)	10.5
SEP	45	2	0	27	16	0.07 (0.01–0.23)	1.00 (0.79-1.00)	1.00 (0.16-1.00)	0.37 (0.23–0.53)	0
NSE	97	11	0	54	32	0.17 (0.09–0.28)	1.00 (0.89-1.00)	1.00 (0.72-1.00)	0.37 (0.27–0.48)	0
**Assessments ≤ 14 after CA**										
PLR + CR	98	16	1	49	32	0.25 (0.15–0.37)	0.97 (0.84-1.00)	0.94 (0.71-1.00)	0.40 (0.29–0.51)	3
EEG	75	24	0	31	20	0.44 (0.30–0.58)	1.00 (0.83-1.00)	1.00 (0.86-1.00)	0.39 (0.26–0.54)	0
SEP	61	3	0	38	20	0.07 (0.02–0.20)	1.00 (0.83-1.00)	1.00 (0.29-1.00)	0.34 (0.22–0.48)	0
NSE	100	7	0	60	33	0.10 (0.04–0.20)	1.00 (0.89-1.00)	1.00 (0.59-1.00)	0.35 (0.26–0.46)	0
**Assessments ≤ 7 after CA**										
EEG + NSE	58	7	0	33	18	0.17 (0.07–0.33)	1.00 (0.81-1.00)	1.00 (0.59-1.00)	0.35 (0.22–0.50)	0
EEG + PLR+CR	56	8	1	30	17	0.21 (0.10–0.37)	0.94 (0.73-1.00)	0.89 (0.52-1.00)	0.36 (0.23–0.51)	5.6
EEG + SEP	41	2	0	24	15	0.08 (0.01–0.25)	1.00 (0.78-1.00)	1.00 (0.16-1.00)	0.38 (0.23–0.55)	0
PLR + CR + NSE	82	7	0	47	28	0.13 (0.05–0.25)	1.00 (0.88-1.00)	1.00 (0.59-1.00)	0.37 (0.26–0.49)	0
PLR + CR + SEP	43	1	0	27	15	0.04 (0.00-0.18)	1.00 (0.78-1.00)	1.00 (0.03-1.00)	0.36 (0.22–0.52)	0
SEP + NSE	45	1	0	28	16	0.03 (0.00-0.18)	1.00 (0.79-1.00)	1.00 (0.03-1.00)	0.36 (0.22–0.52)	0
**Assessments ≤ 14 after CA**										
EEG + NSE	75	5	0	50	20	0.09 (0.03–0.20)	1.00 (0.83-1.00)	1.00 (0.48-1.00)	0.29 (0.18–0.41)	0
EEG + PLR+CR	74	10	0	44	20	0.19 (0.09–0.31)	1.00 (0.83-1.00)	1.00 (0.69-1.00)	0.31 (0.20–0.44)	0
EEG + SEP	56	3	0	34	19	0.08 (0.02–0.22)	1.00 (0.82-1.00)	1.00 (0.29-1.00)	0.36 (0.23–0.50)	0
PLR + CR + NSE	97	3	0	61	33	0.05 (0.01–0.13)	1.00 (0.89-1.00)	1.00 (0.29-1.00)	0.35 (0.26–0.46)	0
PLR + CR + SEP	59	0	0	39	20	0.00 (0.00-0.09)	1.00 (0.83-1.00)	NA	0.34 (0.22–0.47)	0
SEP + NSE	61	1	0	40	20	0.02 (0.00-0.13)	1.00 (0.83-1.00)	1.00 (0.03-1.00)	0.33 (0.22–0.47)	0

For each single and paired marker, number of patients with available measurements are presented in n, using the last available measurement per predictor and patient within the window ≤ 7 (upper part) and ≤ 14 (lower part) days. Results presented in numbers or in percentages with 95% confidence intervals (CI). TP + FN equals the number of patients with poor outcome at 12 months. FP + TN equals the number of patients with good outcome at 12 months. TP, true positive (predicted and reported poor outcome); TN, true negative (predicted and reported good outcome); FP, false positive (predicted poor outcome, reported good outcome); FN, false negative (predicted good outcome, reported poor outcome); PPV, Positive predictive value; NPV, Negative predictive value; FPR, False Positive Rate

Repeated marker assessments were common within 14 days for PLR + CR, EEG, and NSE, but uncommon for SEP. Using the initial instead of the last available result substantially increased FPRs for PLR + CR (3.0% to 18.2%) and EEG (0% to 10.0%), whereas SEP and NSE showed no FPRs regardless of assessment timing; Supplementary Table S2.

Sensitivity analysis to compare the marker profiles of WLST-excluded patients with the two outcome subgroups of the analyzed cohort showed that WLST patients were clinically indistinguishable from both subgroups at admission (all baseline characteristics *p* ≥ 0.07, Bonferroni-corrected). However, within the first two weeks their prognostic profile diverged markedly: on every measured marker, WLST patients differed significantly from patients who recovered, while their marker burden was equivalent to or exceeded that of patients who had documented poor outcomes under continued care (NSE median 81.6 vs. 29.6 vs. 20.3 µg/L within 14 days; absent SEP 48% vs. 7% vs. 0%; CRS-R best 1.0 vs. 4.0 vs. 17.0; Supplementary Table S3). Among 37 WLST patients with available death dates, median time from CA to death was 11 (7–17) days.

### Outcome prediction according to prognostic classification

Of 101 patients, 86 (85.1%; ≤7-day window) and 99 (98.0%; ≤14-day window) had ≥ 2 different measured markers and were included in the respective analyses; excluded patients lacked a second marker almost exclusively because EEG or SEP was unavailable or non-evaluable. Included and excluded patients did not differ in any baseline characteristic (all *p* ≥ 0.23 and ≥ 0.40, respectively) or in 12-month outcome (Fisher exact *p* = 0.37 and *p* = 1.0). In age-adjusted Firth logistic regression, including patients with at least two measured markers, one unfavorable marker within 7 days (*N* = 86) was not associated with poor outcome (*n* = 18; aOR 2.12, 95% CI 0.71–7.11, *p* = 0.184), whereas ≥ 2 unfavorable markers were (*n* = 15; aOR 7.18, 95% CI 1.57–69.0, *p* = 0.0086), compared with the 0-marker category (*n* = 53). FP classifications occurred in five patients with one unfavorable marker and in one patient with ≥ 2 unfavorable markers. In the latter, the ≥ 2-marker classification was driven by unfavorable EEG and PLR + CR, whereas SEP was present and NSE was elevated but below the predefined cut-off (44.7 µg/L); by the second week, no unfavorable markers remained in this patient.

Within 14 days (*N* = 99), both one unfavorable marker (*n* = 16; aOR 15.4, 95% CI 3.19–154, *p* = 0.00019) and ≥ 2 unfavorable markers (*n* = 16; aOR 34.7, 95% CI 4.11–4578, *p* = 0.00010) were associated with poor outcome compared with the 0-marker category (*n* = 67). At this later time point, one FP case remained in the 1-marker category, whereas no patient with ≥ 2 unfavorable marker had a good outcome. The unbounded upper confidence limits in the 14-day analysis and, particularly for the ≥ 2-marker stratum, reflects quasi-complete separation of outcome by exposure (0/16 good outcomes). In this setting, the penalized likelihood ratio test p-values reflect strong evidence against the null of no association, while the wide upper confidence bounds reflect limited information to constrain the magnitude of the effect rather than uncertainty about its direction or clinical relevance.

Sensitivity analyses assigning all lost to follow-up patients an entirely favorable or entirely unfavorable 12-month outcome yielded aORs for ≥ 2 unfavorable markers within 14 days between 26.3 and 41.1, bracketing the primary estimate of 34.7; FPRs for EEG, SEP, and NSE within 14 days remained 0% under both extremes; Supplementary Table S4.

### CRS-R scores improve prognostic performance

**Fig. 2:**
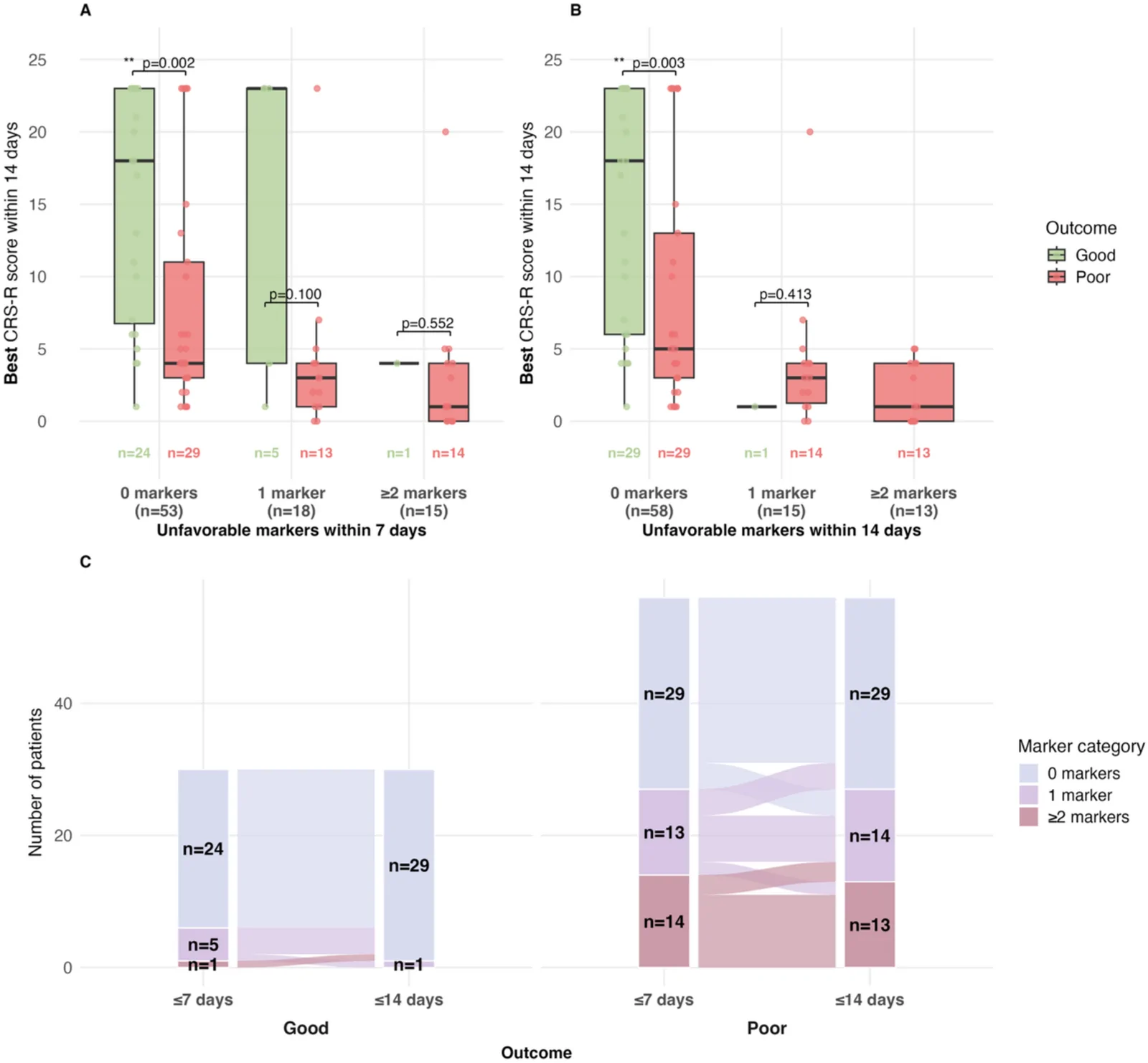
Best CRS-R score within 14 days by marker category and marker category transitions between week one and week two. Panels **A** and **B** show the distribution of the best CRS-R score achieved within 14 days after cardiac arrest, stratified by the number of unfavorable markers (0, 1, or ≥ 2) assessed within 7 days (A) and 14 days (B), separately for patients with good (mRS 0–3) and poor outcome (mRS 4–6). Only patients with at least two markers measured within the first 7 days were included. Horizontal bars indicate pairwise comparisons between outcome groups within each marker category (Wilcoxon rank-sum test; * *p* < 0.05, ** *p* < 0.01, *** *p* < 0.001). Panel **C** illustrates the transition of individual patients across marker burden categories from week 1 (≤ 7 days) to week 2 (≤ 14 days), based on the last available measurement within each time window. The width of each flow is proportional to the number of patients transitioning between categories. Numbers within strata indicate patient counts. CRS-R, Coma Recovery Scale–Revised; mRS, modified Rankin Scale

Initial CRS-R scores were low across all marker categories and did not differ between good and poor outcome groups (0 markers: median 1.0 [IQR 0.0–3.5] vs. 2.0 [1.0–5.0]; 1 marker: 1.0 [0.0–1.0] vs. 1.0 [0.0–3.0]; ≥2 markers: 0.0 [0.0–0.0] vs. 0.0 [0.0–2.5]). Best CRS-R scores within 14 days differed by marker category. Patients with good outcome and 0 unfavorable markers within 7 days achieved higher CRS-R scores compared to those with poor outcome; Fig. 2A.

Marker categories were largely stable between weeks 1 and 2 for patients with poor outcome, while most patients with good outcome remained in or shifted to the 0-marker category; Fig. 2C. Among good outcome patients in the 1-marker category at ≤ 7 days, best CRS-R scores were comparable to those in the 0-marker category, but not when markers were reassessed at ≤ 14 days; Fig. 2A & B. Transition analysis showed that 4 of these 5 patients shifted to the 0-marker, suggesting that both favorable marker and high CRS-R during the second week indicate good outcome, Fig. 2C.

Within the entire study population, the best CRS-R score showed moderate discrimination for poor outcome (AUC 0.769 [95% CI 0.669–0.869]). At a required specificity of ≥ 0.90, the most sensitive cut-offs were CRS-*R* ≤ 3 for poor outcome (sensitivity 46.3%, FPR 9.1%) and CRS-*R* ≥ 21 for good outcome (sensitivity 39.4%, FPR 9.0%). Application of this CRS-R thresholds provided incremental classification beyond marker category; Table 3. The poor outcome threshold performed with high precision across all marker categories (88.9–100%), offering the greatest net gain in patients with 0 or 1 marker, where marker burden alone was insufficient for clear prognosis. The good outcome threshold was less reliable, with lower precision and no patient in the ≥ 2-marker category reaching the threshold.

**Table 3:** Incremental classification by CRS-R thresholds across unfavorable marker categories within 7 days after CA

	0 markers	1 marker	≥ 2 markers
**Poor outcome — CRS- ≤ 3**			
n true Poor/N category	29/53	13/18	14/15
CRS-R classifies as Poor, n	10	9	9
Correct classifications, n	9	8	9
False classifications, n	1	1	0
Precision, %	90.0	88.9	100.0
Net gain, n	8	7	9
**Good outcome — CRS-R ≥ 21**			
n true Good/N category	24/53	5/18	1/15
CRS-R classifies as Good, n	15	4	0
Correct classifications, n	10	3	0
False classifications, n	5	1	0
Precision, %	66.7	75.0	NA
Net gain, n	5	2	0

CRS-R, Coma Recovery Scale–Revised; n, number of patients; N, number of total patients per marker burden category. Unfavorable markers assessed within 7 days. Net gain = correct − false classifications. Precision = correct classifications / total classified by CRS-R threshold. In the ≥ 2-marker category, poor outcome was already indicated by marker burden alone; CRS-R classification in this group does not represent true incremental prognostic value

### Multivariable models within 14 days

In multivariable Firth logistic regression, SEP was excluded because of high missing (*n* = 42) and low unfavorable findings (*n* = 3). In the CC analysis (*N* = 74; poor outcome *n* = 54), the baseline model including centered age, EEG, PLR + CR, and NSE (scaled per 10 µg/L) showed good discrimination (apparent AUC 0.912, optimism-corrected AUC 0.898, cross-validated AUC 0.888) with a Brier score of 0.109, calibration intercept of 0.112, and calibration slope of 1.32. Older age (OR 1.07 per year, 95% CI 1.02–1.14, *p* = 0.0056), unfavorable EEG (OR 26.9, 95% CI 2.84–3640, *p* = 0.0015), absent PLR + CR (OR 25.4, 95% CI 1.94–3880, *p* = 0.0098), and higher NSE (OR 1.47, 95% CI 1.01–2.55, *p* = 0.041) were independently associated with poor outcome.

Adding best CRS-R improved model performance (apparent AUC 0.953, optimism-corrected AUC 0.936, cross-validated AUC 0.925) and reduced the apparent and cross-validated Brier scores to 0.084 and 0.104, and left calibration similar (intercept 0.122; slope 1.31). In the extended model, older age (OR 1.09 per year, 95% CI 1.03–1.16, *p* = 0.0020), unfavorable EEG (OR 13.0, 95% CI 1.22–1810, *p* = 0.0308), absent PLR + CR (OR 19.0, 95% CI 1.08–3250, *p* = 0.0434), higher NSE (OR 1.48, 95% CI 1.04–2.59, *p* = 0.0297) were independently associated with poor outcome. For CRS-R, lower scores were associated with poor outcome (OR 1.13 per 1-point decrease, 95% CI 1.03–1.27, *p* = 0.0073), and addition of CRS-R improved discrimination and overall model fit (LR test *p* = 0.011). A 2 × 2 sensitivity analysis varying the CRS-R measurement window and the survival restriction showed that the CRS-R coefficient was robust to survival restriction within both windows (7-day window: full cohort: OR 1.006, *p* = 0.95; restricted: OR 1.011, *p* = 0.91; 14-day window: full cohort: OR 0.884, *p* = 0.007 [primary]; restricted: OR 0.891, *p* = 0.012).

Applied to the 30 WLST patients with complete predictor data, the multivariable Firth model (with CRS-R scores) yielded a median predicted probability of 0.999 (IQR 0.987–1.000), with predicted probability ≥ 0.5 in 29 of 30 patients (96.7%), ≥ 0.8 in 29 of 30 (96.7%), and ≥ 0.9 in 27 of 30 (90.0%); Supplementary Table S5. All three WLST patients with predicted P(poor outcome) < 0.9 (*P* = 0.20, 0.81, 0.83) had favorable EEG, intact PLR, and low NSE, alongside substantial non-neurological comorbidity, with significantly higher mCIRS subscores than the analyzed cohort for hepatic, renal, urogenital, and pre-existing neurological involvement (all *p* ≤ 0.040); the youngest (36 years) survived 90 days despite a poor-outcome prediction of only 20%, while the other two (68 and 73 years) had multi-system disease with pre-existing neurological conditions. This combination of reassuring neurological markers and high non-neurological comorbidity burden is more consistent with WLST driven by multi-system disease than with premature withdrawal of patients with retained recovery potential, though the specific reasons for individual WLST decisions are beyond the scope of this analysis.

## Discussion

In this prospective multicenter study of patients who remained comatose for at least 3 days after CA and in which patients with early WLST (< 4 weeks) were excluded, we observed only one single patient with two unfavorable markers (PLR + CR and EEG) and good outcome. The results show a severely affected group of patients, more than two-thirds of whom had poor outcomes (mRS 4–6) at 12 months and a mortality rate of 43%. Although the WLST exclusion might shift the severity distribution by removing its most extreme tail, the analyzed cohort still captures the full range of outcomes from full recovery to death and is therefore representative of comatose post-arrest patients under continued care rather than of a milder selected population.

Prognostication of long-term poor outcome was more accurate when based on ≥ 2 unfavorable markers rather than single markers, which is in line with current guidelines [6–8]. Prognostic accuracy increased over time, and repeated assessment reduced FP classifications, particularly for PLR + CR and EEG. The CRS-R provided additional prognostic information, especially in patients with uncertain prognosis according to current guidelines (0–1 unfavorable markers) and remained independently associated with poor outcome in multivariable modeling.

A major challenge in neuroprognostication after CA is the risk of self-fulfilling prophecies introduced by WLST [5]. Previous studies have addressed this through blinded assessment [23], exclusion of index predictors from treatment decisions [24], or structured independent prognostic procedures [25]. In contrast, the present study addressed this in two ways. First, we excluded patients with early (< 4 weeks) WLST from the primary analysis, removing the most direct mechanism by which prognostic findings could influence the observed outcome. Second, in a sensitivity analysis we compared the marker profiles of WLST-excluded patients with the two outcome subgroups of the analyzed cohort. WLST patients were clinically indistinguishable from the analyzed cohort at enrollment on all baseline and initial neurological measures, indicating that early treatment-limitation decisions were not driven by overt baseline differences. Within the first two weeks, however, their prognostic profile diverged sharply from patients who recovered, with marker burden equivalent to or exceeding that of patients who had documented poor outcomes under continued care. Notably, no measured marker showed WLST patients resembling the good-outcome subgroup - the pattern that premature treatment limitation in recoverable patients would produce. While residual influence of marker findings on later care decisions cannot be fully excluded, these results reduce the possibility of residual self-fulfilling-prophecy bias in our cohort. The multivariable model, refit on the analyzed cohort, predicted poor outcome with probability ≥ 0.9 in 90% of WLST patients with complete predictors; the three with lower predicted probabilities had a substantially higher burden of pre-existing hepatic, renal, urogenital, and neurological comorbidities, consistent with non-neurological reasons for treatment limitation. This analysis does not validate model accuracy against WLST outcomes as death following WLST is uninformative for that purpose but characterizes WLST patients’ marker profiles using a model trained on a cohort in which early WLST was excluded. Under the assumption that the marker–outcome relationship reflects biology rather than treatment effects, the near-certain poor-outcome predictions in WLST patients indicate that their marker profiles match those patients with observed poor outcomes under continued care. This is the opposite of what premature limitation in recoverable patients would produce. Our findings therefore can be generalize to comatose post-CA patients managed with continued care for at least four weeks.

Our finding of 0% FPR for ≥ 2 unfavorable markers within 14 days replicates results from the two largest cohorts in which the self-fulfilling-prophecy mechanism was minimized by clinical context rather than by study design: the KORHN registry from South Korea [26] and the ProNeCA prospective multicenter study from Italy in which WLST was not performed [27]. Both reported 100% specificity for every recommended pairwise combination of guideline markers. The convergence of three independently designed WLST-controlled cohorts on the same specificity finding strengthens the inference that multimodal prognostication can achieve 0% FPR independent of self-fulfilling-prophecy bias.

Within single marker assessments FPRs for PLR + CR and EEG have been previously reported [28, 29]. For PLR + CR, our manual assessment may have contributed to this limitation. Quantitative pupillometry has been shown to reduce FP findings [23, 30]. Similarly, the prognostic specificity of highly malignant EEG patterns may depend on strict acquisition and interpretation standards and could potentially be improved by systematic incorporation of additional features such as reactivity [31]. For NSE, the chosen cut-off (≥ 90 µg/L) reached high specificity, as shown previously [32] but identified less than 25% of poor-outcome patients. This higher threshold was chosen to prioritize specificity over sensitivity, in line with a multicenter study that showed that a cutoff of 90 µg/L yields a 0% FPR for poor outcome compared with a residual FPR at 60 µg/L.^32^ For SEP, the absence of FP predictions in our cohort is consistent with previous reports [33, 34], but the very small number of unfavorable SEP findings might limit the robustness of this observation. Our systematic re-evaluation identified 14 recordings from 11 patients as non-evaluable due to high noise level and no reliably reproduced peripheral/spinal potentials. This underscores the need for strict SEP quality standards in CA patients, particularly regarding bilateral replication of potentials and noise levels of recordings [20].

Importantly, FP classifications were largely confined within 7 days and were no longer observed in patients with ≥ 2 unfavorable markers by 14 days. In contrast, fewer than one quarter of patients with poor outcomes in our CA cohort were identified by these criteria. The trade-off between low FPRs on the cost of sensitivity is well known, with sensitivity ranging from 18% to 60%.^29,35^ The comparatively lower sensitivity observed in our analyzed cohort reflects exclusion of the most extreme tail of HIBI severity through the early-WLST exclusion criterion, rather than enrichment for mildly injured patients. The analyzed cohort itself remained predominantly severely affected, with 68% of patients experiencing poor 12-month outcome (mRS 4–6) including 43% mortality.

Our repeated assessment reduced FP classifications, whereas reliance on the initial finding increased FPRs. This observation is clinically relevant as WLST is frequently performed between 3 and 10 days after CA [14, 15] while delayed recovery of consciousness up to two weeks is well documented [16, 17].

An important finding in our cohort of patients with a high proportion of uncertain prognosis according to standard neuroprognostication was the added prognostic value of structured behavioral assessment. The CRS-R is not proposed as an independent prognostic biomarker comparable to EEG, SEP, NSE, or PLR + CR; rather, it is a structured behavioral-recovery instrument positioned as a complementary indicator in the subgroup of patients whose prognosis remains indeterminate after multimodal biomarker testing (0 or 1 unfavorable markers). The initial CRS-R score within 14 days was not informative, whereas the best CRS-R score was associated with outcome and improved prognostic discrimination beyond age, EEG, PLR + CR, and NSE. Its added value was most apparent in patients with 0–1 unfavorable markers, in whom the CRS-R further separated good from poor outcomes. The CRS-R’s prognostic value was time-dependent, reflecting the course of behavioral recovery rather than differential survival opportunity, and indicating that prognostically meaningful behavioral signs do not reliably emerge before the second week post-cardiac arrest. Although the data-derived threshold of CRS-*R* ≤ 3 may be clinically useful as a pragmatic red-flag criterion, the continuous CRS-R score provided stronger incremental prognostic information in multivariable modeling. Consistent with its established role in neurorehabilitation [10, 11], the CRS-R may also have an important role in early neuroprognostication by complementing established markers. We do not propose it as a substitute for any guideline-recommended modality.

Several limitations should be considered. Excluding patients with early WLST unavoidably introduces selection bias. Among patients with comparable prognostic profiles, the decision to withdraw or continue care might be driven by factors other than the prognostic markers, including documented or presumed patient will, premorbid functional status, family preference, and non-neurological comorbidity burden. Our findings therefore generalize to comatose post-CA patients managed with continued care for at least four weeks, and our sensitivity analyses reduce but do not fully eliminate the possibility of residual self-fulfilling-prophecy bias. The sample size was modest, and the CC multivariable analysis included only 74 patients, resulting in wide confidence intervals for some predictors. Missingness remained substantial for some markers, particularly SEP, which limited precision and restricted combined modeling. Given the near-complete separation in the group with ≥ 2 markers, point estimates of the effect size should be interpreted with caution; however, the direction, statistical significance and observed FPRs remain robust. Nine patients (8.2%) were excluded due to missing 12-month mRS, they did not differ from the analyzed cohort on any measured characteristic, and sensitivity analyses with extreme outcome imputation preserved the marker performance findings. Outcome assessors were blinded to predictor results in all but one center, where the same researchers conducted baseline and follow-up assessments; the function-based nature of the mRS limits but does not eliminate residual assessor bias. PLR + CR was assessed as part of the FOUR score, rather than separately. Neuroimaging was not included and therefore the full spectrum of guideline-recommended prognostic tools could not be assessed. Cause of death and downstream treatment-limitation decisions were not captured systematically; while published evidence indicates that WLST is typically an early-ICU decision, with a median time from CA to WLST of approximately 143 hours [36], we cannot fully exclude that later, more subtle care decisions influenced by neuroprognostic results contributed to mortality in our cohort. Dichotomization of the mRS, while standard in this literature, obscures distinctions within outcome categories such as severe disability versus death, or full recovery versus mild impairment. Finally, model performance was assessed by internal but not external validation.

The study has important strengths. In contrast to many retrospective analyses [29, 33, 35], our prospective observational approach ensured standardized assessments, a clear temporal relationship between prognostic results and long-term outcome, and reduced confounding by including only patients who remained comatose three days after CA. Comparison of initial and repeated marker assessments provides clinically relevant information on temporal stability, and the multimethod analytical approach supports the robustness of the CRS-R findings.

## Conclusion

Overall, in this cohort of patients who remained comatose for at least three days after CA and were followed without WLST within the first 28 days, multimodal prognostication identified poor 12-month outcome with high specificity, consistent with findings from other WLST-controlled cohorts. Reassessment of initially unfavorable findings reduced FP classifications, and the CRS-R provided complementary prognostic information in patients with indeterminate marker profiles. Although self-fulfilling-prophecy bias cannot be fully excluded in any observational prognostication cohort, the marker profiles of WLST-excluded patients within the first four weeks matched or exceeded those of patients with documented poor outcomes and did not resemble those of recoverable patients, which reduces - though it does not completely rule it out - the likelihood that such bias is the primary reason for the observed associations. The direction of the findings is robust, even though the exact effect size could not be precisely quantified given the cohort size; external validation in larger cohorts is required to refine these estimates.

## Supplementary Information

Below is the link to the electronic supplementary material.

Supplementary Material 1

Supplementary Material 2

Supplementary Material 3

Supplementary Material 4

Supplementary Material 5

Supplementary Material 6

## Data Availability

Pseudonymized participant data and a data dictionary underlying the results reported in this article can be made available to researchers in anonymized form upon request.

## Abbreviations

aOR: Adjusted Odds Ratio
AUC: Area Under the Curve
BI: Barthel Index
CA: Cardiac Arrest
CC: Complete-Case
CI: Confidence Interval
CR: Corneal Reflex
CRS-R: Coma Recovery Scale–Revised
DoC: Disorders of Consciousness
FN: False Negative
FOUR: Full Outline of UnResponsiveness
FP: False Positive
FPR: False Positive Rate
GCS: Glasgow Coma Scale
HIBI: Hypoxic-Ischemic Brain Injury
ICU: Intensive Care Unit
IHCA: In-Hospital Cardiac Arrest
IQR: Interquartile Range
LOS: Length of Stay
mCIRS: Modified Cumulative Illness Rating Scale
mRS: Modified Rankin Scale
NPV: Negative Predictive Value
NSE: Neuron-Specific Enolase
OHCA: Out-of-Hospital Cardiac Arrest
OR: Odds Ratio
PLR: Pupillary Light Reflex
PPV: Positive Predictive Value
ROC: Receiver Operating Characteristic
ROSC: Restoration of Spontaneous Circulation
SD: Standard Deviation
SEP: Somatosensory Evoked Potential
TN: True Negative
TP: True Positive
WLST: Withdrawal of Life-Sustaining Therapy
