# Supplementary material for: Neuroprognostication after cardiac arrest in patients without withdrawal of life-sustaining therapy: a prospective observational multicenter study
Source: Crit Care. 2026 Jul 24;30:391. doi: 10.1186/s13054-026-06209-0 (PMC13404381; doi:10.1186/s13054-026-06209-0)
Supplement: Supplementary file 2 — Supplementary Material 2 [file 13054_2026_6209_MOESM2_ESM.docx]

**Supplementary Material**

**Supplementary Table S1**

Table S1 Outcome measures including assessments and time points of data collection

| Variable | Assessment | Screening | Week one | Week two | Follow-up |
| --- | --- | --- | --- | --- | --- |
| **Primary outcome** | **mRS** |  |  |  | **x** |
| Demographic data  Age  Gender |  | x  x |  |  |  |
| Clinical data  Location of CA  Cause of CA  Use of TTM |  | x  x  x |  |  |  |
| Comorbidities | mCIRS |  | x |  |  |
| Neurological status | GCS, CRS-R, FOUR | x | x | x | x |
| Prognostic markers | PLR+CR, EEG, SEP, NSE |  | x | x |  |
| Independence | BI |  |  |  | x |

BI, Barthel Index; CRS-R, Coma Recovery Scale–Revised; FOUR, Full Outline of UnResponsiveness score; GCS, Glasgow Coma Scale; mCIRS, modified Cumulative Illness Rating Scale; mRS, modified Rankin Scale; NSE, neuron-specific enolase; PLR+CR, pupillary light and corneal reflex; SEP, somatosensory evoked potential (N20 wave); TTM, targeted temperature management
