# Supplementary material for: Neuroprognostication after cardiac arrest in patients without withdrawal of life-sustaining therapy: a prospective observational multicenter study
Source: Crit Care. 2026 Jul 24;30:391. doi: 10.1186/s13054-026-06209-0 (PMC13404381; doi:10.1186/s13054-026-06209-0)
Supplement: Supplementary file 3 — Supplementary Material 3 [file 13054_2026_6209_MOESM3_ESM.docx]

Table S2 Prognostic performance of initial single and paired prognostic markers within 7 and 14 days after CA

| Prognostic marker (single and paired) | n | TP | FP | FN | TN | Sensitivity (95% CI) | Specificity (95% CI) | PPV  (95% CI) | NPV  (95% CI) | FPR (%) |
| --- | --- | --- | --- | --- | --- | --- | --- | --- | --- | --- |
| Assessments ≤7 after CA | | | | | | | | | | |
| PLR+CR | 83 | 19 | 6 | 35 | 23 | 0.35 (0.23–0.49) | 0.79 (0.60–0.92) | 0.76 (0.55–0.91) | 0.40 (0.27–0.53) | 20.7 |
| EEG | 59 | 17 | 2 | 23 | 17 | 0.42 (0.27–0.59) | 0.89 (0.67–0.99) | 0.89 (0.67–0.99) | 0.42 (0.27–0.59) | 10.5 |
| SEP | 45 | 2 | 0 | 27 | 16 | 0.07 (0.01–0.23) | 1.00 (0.79–1.00) | 1.00 (0.16–1.00) | 0.37 (0.23–0.53) | 0 |
| NSE | 97 | 12 | 0 | 53 | 32 | 0.18 (0.10–0.30) | 1.00 (0.89–1.00) | 1.00 (0.74–1.00) | 0.38 (0.27–0.49) | 0 |
| Assessments ≤14 after CA | | | | | | | | | | |
| PLR+CR | 98 | 26 | 6 | 39 | 27 | 0.40 (0.28–0.53) | 0.82 (0.65–0.93) | 0.81 (0.64–0.93) | 0.41 (0.29–0.54) | 18.2 |
| EEG | 75 | 25 | 2 | 30 | 18 | 0.45 (0.32–0.59) | 0.90 (0.68–0.99) | 0.93 (0.76–0.99) | 0.38 (0.24–0.53) | 10.0 |
| SEP | 61 | 3 | 0 | 38 | 20 | 0.07 (0.02–0.20) | 1.00 (0.83–1.00) | 1.00 (0.29–1.00) | 0.34 (0.22–0.48) | 0 |
| NSE | 100 | 12 | 0 | 55 | 33 | 0.18 (0.10–0.29) | 1.00 (0.89–1.00) | 1.00 (0.74–1.00) | 0.38 (0.27–0.48) | 0 |
| Assessments ≤7 after CA | | | | | | | | | | |
| EEG + NSE | 58 | 7 | 0 | 33 | 18 | 0.17 (0.07–0.33) | 1.00 (0.81–1.00) | 1.00 (0.59–1.00) | 0.35 (0.22–0.50) | 0 |
| EEG + PLR+CR | 56 | 8 | 1 | 30 | 17 | 0.21 (0.10–0.37) | 0.94 (0.73–1.00) | 0.89 (0.52–1.00) | 0.36 (0.23–0.51) | 5.6 |
| EEG + SEP | 41 | 2 | 0 | 24 | 15 | 0.08 (0.01–0.25) | 1.00 (0.78–1.00) | 1.00 (0.16–1.00) | 0.38 (0.23–0.55) | 0 |
| PLR+CR + NSE | 82 | 7 | 0 | 47 | 28 | 0.13 (0.05–0.25) | 1.00 (0.88–1.00) | 1.00 (0.59–1.00) | 0.37 (0.26–0.49) | 0 |
| PLR+CR + SEP | 43 | 1 | 0 | 27 | 15 | 0.04 (0.00–0.18) | 1.00 (0.78–1.00) | 1.00 (0.03–1.00) | 0.36 (0.22–0.52) | 0 |
| SEP + NSE | 45 | 1 | 0 | 28 | 16 | 0.03 (0.00–0.18) | 1.00 (0.79–1.00) | 1.00 (0.03–1.00) | 0.36 (0.22–0.52) | 0 |
| Assessments ≤14 after CA | | | | | | | | | | |
| EEG + NSE | 75 | 9 | 0 | 46 | 20 | 0.16 (0.08–0.29) | 1.00 (0.83–1.00) | 1.00 (0.66–1.00) | 0.30 (0.20–0.43) | 0 |
| EEG + PLR+CR | 74 | 15 | 1 | 39 | 19 | 0.28 (0.16–0.42) | 0.95 (0.75–1.00) | 0.94 (0.70–1.00) | 0.33 (0.21–0.46) | 5.0 |
| EEG + SEP | 56 | 3 | 0 | 34 | 19 | 0.08 (0.02–0.22) | 1.00 (0.82–1.00) | 1.00 (0.29–1.00) | 0.36 (0.23–0.50) | 0 |
| PLR+CR + NSE | 97 | 10 | 0 | 54 | 33 | 0.16 (0.08–0.27) | 1.00 (0.89–1.00) | 1.00 (0.69–1.00) | 0.38 (0.28–0.49) | 0 |
| PLR+CR + SEP | 59 | 1 | 0 | 38 | 20 | 0.03 (0.00–0.13) | 1.00 (0.83–1.00) | 1.00 (0.03–1.00) | 0.34 (0.22–0.48) | 0 |
| SEP + NSE | 61 | 1 | 0 | 40 | 20 | 0.02 (0.00–0.13) | 1.00 (0.83–1.00) | 1.00 (0.03–1.00) | 0.33 (0.22–0.47) | 0 |

**Note.** For each single and paired marker, number of patients with available measurements are presented in n, using the initial measurement per predictor and patient within the window ≤7 (upper part) and ≤14 (lower part) days. Results presented in numbers or in percentages with 95% confidence intervals (CI). TP + FN equals the number of patients with poor outcome at 12 months. FP + TN equals the number of patients with good outcome at 12 months. TP, true positive (predicted and reported poor outcome); TN, true negative (predicted and reported good outcome); FP, false positive (predicted poor outcome, reported good outcome); FN, false negative (predicted good outcome, reported poor outcome); PPV, Positive predictive value; NPV, Negative predictive value; FPR, False Positive Rate
