# Supplementary material for: Neuroprognostication after cardiac arrest in patients without withdrawal of life-sustaining therapy: a prospective observational multicenter study
Source: Crit Care. 2026 Jul 24;30:391. doi: 10.1186/s13054-026-06209-0 (PMC13404381; doi:10.1186/s13054-026-06209-0)
Supplement: Supplementary file 4 — Supplementary Material 4 [file 13054_2026_6209_MOESM4_ESM.docx]

Table S5 Model-predicted probability of poor outcome for WLST patients with complete predictors.

| **Patient** | **Age**  **(years)** | **mCIRS** | **EEG** | **PLR/CR** | **NSE**  **(µg/L)** | **CRS-R**  **best** | **Survival**  **(days)** | **Predicted P**  **(poor outcome)** |
| --- | --- | --- | --- | --- | --- | --- | --- | --- |
| 1 | 47 | 9 | favorable | absent | 15.3 | 1 | 17 | 0.9202 |
| 2 | 84 | 8 | favorable | absent | 318.3 | 0 | 5 | >0.999 |
| 3 | 55 | NA | unfavorable | absent | 40.7 | 1 | 16 | 0.9987 |
| 4 | 71 | 17 | favorable | intact | 63.8 | 3 | 18 | 0.9592 |
| 5 | 72 | 7 | unfavorable | absent | 218.0 | 1 | 17 | >0.999 |
| 6 | 61 | 14 | unfavorable | absent | 108.0 | 0 | 16 | >0.999 |
| 7 | 71 | 12 | unfavorable | absent | 305.0 | 2 | 12 | >0.999 |
| 8 | 74 | 9 | unfavorable | absent | 38.9 | 1 | 10 | 0.9997 |
| 9 | 58 | 8 | unfavorable | absent | 88.4 | 0 | 9 | >0.999 |
| 10 | 70 | 1 | unfavorable | intact | 100.0 | 0 | 7 | 0.9994 |
| 11 | 42 | 12 | unfavorable | absent | 81.6 | 0 | 4 | 0.9993 |
| 12 | 69 | 2 | unfavorable | absent | 784.0 | 1 | 6 | >0.999 |
| 13 | 67 | 12 | unfavorable | absent | 49.3 | 0 | 24 | 0.9997 |
| 14 | 70 | 15 | unfavorable | intact | 20.2 | 1 | 11 | 0.9849 |
| 15 | 75 | 12 | unfavorable | absent | 513.0 | 0 | 6 | >0.999 |
| 16 | 58 | 0 | favorable | intact | 202.0 | 3 | 9 | 0.9994 |
| 17 | 75 | 6 | unfavorable | intact | 135.0 | 4 | 29 | 0.9998 |
| 18 | 57 | 0 | favorable | absent | 58.0 | 1 | 11 | 0.9930 |
| 19 | 77 | 15 | unfavorable | absent | 417.0 | 0 | 7 | >0.999 |
| 20 | 69 | 9 | unfavorable | absent | 32.3 | 0 | 11 | 0.9995 |
| 21 | 61 | 5 | unfavorable | intact | 128.0 | 5 | 227 | 0.9992 |
| 22 | 55 | 7 | unfavorable | intact | 119.0 | 2 | 7 | 0.9987 |
| 23 | 33 | 7 | unfavorable | intact | 75.4 | 1 | 8 | 0.9623 |
| 24 | 62 | 7 | unfavorable | intact | 105.0 | 1 | 12 | 0.9989 |
| 25 | 74 | 18 | unfavorable | absent | 31.3 | 5 | 15 | 0.9994 |
| 26 | 37 | 4 | unfavorable | intact | 73.4 | 3 | NA | 0.9627 |
| 27 | 70 | 14 | favorable | intact | 119.0 | 1 | 9 | 0.9958 |
| 28 | 68 | 10 | favorable | intact | 17.3 | 0 | 13 | 0.8111 |
| 29 | 73 | 5 | favorable | intact | 24.9 | 5 | NA | 0.8258 |
| 30 | 36 | 16 | favorable | intact | 31.4 | 6 | 90 | 0.1962 |

**Notes.** Each row corresponds to one WLST patient with complete predictors under the extended multivariable model; patient identifiers are sequential and anonymized. Patients 28–30 (shaded) are the three WLST patients with predicted probability of poor outcome < 0.9 under the extended model; patient 30 (P = 0.196) is the youngest and most discrepant case. EEG and PLR/CR are categorized as favorable/unfavorable and intact/absent, respectively, per the predefined thresholds described in the Methods. NSE values are the within-window peak measurement (µg/L). Survival is reported as days from cardiac arrest; *NA* indicates missing or not applicable (e.g., still alive at last follow-up, missing mCIRS). Predicted probabilities > 0.9999 are reported as “>0.999”. WLST = withdrawal of life-sustaining therapy; mCIRS = modified Cumulative Illness Rating Scale; EEG = electroencephalography; PLR = pupillary light reflex; CR = corneal reflex; NSE = neuron-specific enolase; CRS-R = Coma Recovery Scale–Revised.
