# Supplementary material for: Neuroprognostication after cardiac arrest in patients without withdrawal of life-sustaining therapy: a prospective observational multicenter study
Source: Crit Care. 2026 Jul 24;30:391. doi: 10.1186/s13054-026-06209-0 (PMC13404381; doi:10.1186/s13054-026-06209-0)
Supplement: Supplementary file 5 — Supplementary Material 5 [file 13054_2026_6209_MOESM5_ESM.docx]

Table S3 Sensitivity analysis to compare the marker profiles of WLST-excluded patients with the two outcome subgroups of the analyzed cohort

| **Variable** | **WLST**  **(n=39)** | **Poor outcome**  **(n=68)** | **Good outcome (n=33)** | **Omnibus p** | **WLST vs.**  **Poor (pᵇᶠ)** | **WLST vs.**  **Good (pᵇᶠ)** | **Poor vs.**  **Good (pᵇᶠ)** |
| --- | --- | --- | --- | --- | --- | --- | --- |
| ***Baseline characteristics*** | | | | | | | |
| Age at cardiac arrest (years) | 68.0 (55.0–73.0) [n=39] | 65.5 (55.8–75.0) [n=68] | 60.0 (50.0–71.0) [n=33] | 0.257 | >0.999 | >0.999 | 0.302 |
| Sex | 7 (17.9%) female | 23 (33.8%) female | 6 (18.2%) female | 0.126 | 0.350 | >0.999 | 0.476 |
| Cardiac arrest location | 27 (77.1%) OHCA | 43 (71.7%) OHCA | 22 (73.3%) OHCA | 0.840 | >0.999 | >0.999 | >0.999 |
| Cause of cardiac arrest | cardiac: 22 (66.7%); hypoxic: 3 (9.1%); other: 3 (9.1%); unknown: 5 (15.2%) | cardiac: 38 (64.4%); hypoxic: 14 (23.7%); other: 5 (8.5%); traumatic: 1 (1.7%); unknown: 1 (1.7%) | cardiac: 25 (83.3%); hypoxic: 1 (3.3%); other: 2 (6.7%); traumatic: 1 (3.3%); unknown: 1 (3.3%) | 0.032 | 0.153 | 0.889 | 0.204 |
| GCS at admission | 3.0 (3.0–4.0) [n=34] | 3.0 (3.0–6.0) [n=67] | 3.0 (3.0–4.0) [n=30] | 0.068 | 0.101 | >0.999 | 0.459 |
| mCIRS score | 9.0 (5.0–14.0) [n=37] | 8.0 (4.0–11.5) [n=67] | 4.5 (2.0–6.2) [n=32] | 0.003 | >0.999 | 0.004 | 0.012 |
| Targeted temperature management | 16 (76.2%) | 32 (59.3%) | 19 (76.0%) | 0.211 | 0.579 | >0.999 | 0.621 |
| Shockable initial rhythm | 10 (33.3%) | 23 (45.1%) | 16 (66.7%) | 0.049 | >0.999 | 0.082 | 0.272 |
| CPR duration (min) | 20.0 (15.0–25.0) [n=14] | 21.5 (10.8–35.8) [n=32] | 12.0 (9.0–23.0) [n=17] | 0.213 | >0.999 | 0.845 | 0.282 |
| Time CA to ROSC (min) | 25.0 (19.5–31.2) [n=16] | 30.0 (23.5–41.0) [n=26] | 30.5 (15.5–45.0) [n=14] | 0.413 | 0.456 | >0.999 | >0.999 |
| ***Prognostic markers*** | | | | | | | |
| Unfavorable PLR/CR ≤ 7 days | 16 (57.1%) | 19 (35.2%) | 5 (17.2%) | 0.006 | 0.194 | 0.007 | 0.382 |
| Unfavorable PLR/CR ≤ 14 days | 19 (50.0%) | 16 (24.6%) | 1 (3.0%) | <0.001 | 0.032 | <0.001 | 0.028 |
| Unfavorable SEP ≤ 7 days | 7 (43.8%) | 2 (6.9%) | 0 (0%) | 0.002 | 0.017 | 0.020 | >0.999 |
| Unfavorable SEP ≤ 14 days | 10 (47.6%) | 3 (7.3%) | 0 (0%) | <0.001 | 0.002 | 0.001 | >0.999 |
| Unfavorable EEG ≤ 7 days | 15 (65.2%) | 16 (40.0%) | 2 (10.5%) | 0.001 | 0.209 | 0.001 | 0.099 |
| Unfavorable EEG ≤ 14 days | 21 (67.7%) | 24 (43.6%) | 0 (0%) | <0.001 | 0.130 | <0.001 | <0.001 |
| Unfavorable NSE ≤ 7 days | 19 (55.9%) | 11 (16.9%) | 0 (0%) | <0.001 | <0.001 | <0.001 | 0.043 |
| Unfavorable NSE ≤ 14 days | 17 (45.9%) | 7 (10.4%) | 0 (0%) | <0.001 | <0.001 | <0.001 | 0.276 |
| NSE value ≤ 7 days (µg/L) | 102.5 (58.0–185.2) [n=34] | 40.9 (24.4–69.1) [n=65] | 24.2 (17.4–41.2) [n=32] | <0.001 | <0.001 | <0.001 | 0.002 |
| NSE value ≤ 14 days (µg/L) | 81.6 (32.3–132.0) [n=37] | 29.6 (21.1–54.2) [n=67] | 20.3 (17.2–25.8) [n=33] | <0.001 | <0.001 | <0.001 | 0.010 |
| ***CRS-R scores*** | | | | | | | |
| CRS-R, first ≤ 14 days | 1.0 (0.0–1.8) [n=38] | 1.0 (0.0–3.5) [n=67] | 1.0 (0.0–3.0) [n=33] | 0.136 | 0.127 | >0.999 | >0.999 |
| CRS-R, best ≤ 14 days | 1.0 (0.0–2.0) [n=38] | 4.0 (1.0–6.0) [n=67] | 17.0 (5.0–23.0) [n=33] | <0.001 | <0.001 | <0.001 | <0.001 |
| CRS-R, last ≤ 14 days | 1.0 (0.0–2.0) [n=38] | 4.0 (1.0–6.0) [n=67] | 17.0 (5.0–23.0) [n=33] | <0.001 | 0.002 | <0.001 | <0.001 |
| CRS-R, median ≤ 14 days | 1.0 (0.0–2.0) [n=38] | 2.0 (1.0–5.8) [n=67] | 9.5 (3.5–13.0) [n=33] | <0.001 | 0.001 | <0.001 | <0.001 |

**Note.** Continuous variables are reported as median (IQR) [n]; categorical variables as n (%). For binary prognostic markers and dichotomous baseline variables (sex, cardiac arrest location, TTM, shockable rhythm), only the unfavorable/yes category is shown for brevity; the complementary category is implicit. Omnibus tests across the three groups: Kruskal–Wallis for continuous variables and Fisher exact (or chi-square) for categorical variables. Pairwise comparisons used the corresponding two-group tests (Mann–Whitney U or Fisher exact). p^bf^ denotes Bonferroni-corrected p-values across the three pairwise comparisons per variable; values are capped at 1.000 and reported as “>0.999”. Sample sizes per variable may differ from the maximum group n owing to missing data and are given in square brackets for continuous variables. WLST = withdrawal of life-sustaining therapy; PLR = pupillary light reflex; CR = corneal reflex; SEP = somatosensory evoked potential; EEG = electroencephalography; NSE = neuron-specific enolase; CRS-R = Coma Recovery Scale–Revised; mCIRS = modified Cumulative Illness Rating Scale; GCS = Glasgow Coma Scale; CA = cardiac arrest; ROSC = return of spontaneous circulation; OHCA = out-of-hospital cardiac arrest; IHCA = in-hospital cardiac arrest; TTM = targeted temperature management.
