# Supplementary material for: Neuroprognostication after cardiac arrest in patients without withdrawal of life-sustaining therapy: a prospective observational multicenter study
Source: Crit Care. 2026 Jul 24;30:391. doi: 10.1186/s13054-026-06209-0 (PMC13404381; doi:10.1186/s13054-026-06209-0)
Supplement: Supplementary file 6 — Supplementary Material 6 [file 13054_2026_6209_MOESM6_ESM.docx]

Table S4 Sensitivity analysis varying the CRS-R measurement window (best score within 7 days vs. within 14 days) and the survival restriction (full analyzed cohort vs. patients surviving at least 14 days post-CA)

| **Predictor** | **Observed (primary)** | **Best case** | **Worst case** |
| --- | --- | --- | --- |
| ***≤ 7-day window*** | | | |
| *Sample size* | *n = 86* | *n = 94* | *n = 94* |
| 1 unfavorable marker | 2.12 (0.71–7.11)  p = 0.184 | 1.89 (0.69–5.50)  p = 0.220 | 2.00 (0.69–6.54)  p = 0.209 |
| ≥2 unfavorable markers | 7.18 (1.57–68.96)  p = 0.009 | 9.19 (2.04–87.64)  p = 0.002 | 5.98 (1.31–57.25)  p = 0.018 |
| Age (per year) | 1.03 (0.99–1.06)  p = 0.165 | 1.02 (0.98–1.05)  p = 0.284 | 1.03 (0.99–1.06)  p = 0.145 |
| ***≤ 14-day window*** | | | |
| *Sample size* | *n = 99* | *n = 108* | *n = 108* |
| 1 unfavorable marker | 15.37 (3.19–154.4)  p < 0.001 | 10.37 (2.73–58.54)  p < 0.001 | 11.84 (2.58–115.2)  p < 0.001 |
| ≥2 unfavorable markers | 34.71 (4.11–4578)  p < 0.001 | 41.12 (5.01–5376)  p < 0.001 | 26.27 (3.21–3429)  p < 0.001 |
| Age (per year) | 1.05 (1.01–1.10)  p = 0.012 | 1.04 (1.01–1.08)  p = 0.021 | 1.04 (1.01–1.08)  p = 0.021 |

**Notes.** Values shown are odds ratios with 95% confidence intervals (OR [95% CI]) and the corresponding p-value, from age-adjusted Firth-penalized logistic regression models. Confidence intervals are profile penalized likelihood-based and p-values are derived from the penalized likelihood ratio test, as appropriate for sparse-data and (quasi-)separation settings (Firth 1993; Heinze & Schemper 2002). The reference category for marker burden is 0 unfavorable markers. Wide upper confidence bounds, particularly in the ≤ 14-day ≥2-marker stratum, reflect quasi-complete separation of outcome by exposure (no good-outcome patients in this stratum); the small p-values indicate strong evidence against the null of no association, while the wide upper bounds reflect limited information to constrain the magnitude of the effect. Scenario definitions: *Observed* — primary analysis on patients meeting the inclusion criteria (≥2 measured markers within the respective window); *Best case* / *Worst case* — sensitivity analyses incorporating early-WLST patients under alternative outcome assumptions [add specific definitions]. OR = odds ratio; CI = confidence interval; WLST = withdrawal of life-sustaining therapy.
